# Co‐Producing Personalised Discharge Planning: Developing a Toolkit to Improve Caregiver Involvement in Hospital Transitions

**DOI:** 10.1111/hex.70483

**Published:** 2025-11-05

**Authors:** Kathryn McEwan, Tom Sanders, Sue Carr, Peter Van Der Graaf, Susan Jones, Maria Raisa Jessica Aquino, Mitchell James Hogg, Rakhshanda Hameed, Frank Lai, Christina Cooper, Sebastian Potthoff

**Affiliations:** ^1^ School of Communities and Education University of Northumbria at Newcastle Newcastle Upon Tyne UK; ^2^ School of Health and Life Sciences University of Teesside Middlesbrough UK; ^3^ Population Health Sciences Institute Newcastle University Newcastle upon Tyne UK; ^4^ At‐Risk Mental State Service Tyne and Wear NHS Foundation Trust Newcastle Upon Tyne UK

**Keywords:** caregivers, discharge, implementation theory, Normalisation Process Theory, personalised care, person‐centred care, transitional care

## Abstract

**Background:**

Informal caregivers are often expected to support patients transitioning from hospital to home and yet are routinely overlooked in discharge planning. This gap can leave caregivers unprepared and unsupported, contributing to avoidable complications and distress. In response, we co‐designed a personalised discharge planning toolkit to support meaningful conversations between hospital staff and informal caregivers.

**Methods:**

Using a participatory action research approach, we conducted four co‐design workshops with 22 stakeholders, including informal caregivers, healthcare professionals and voluntary sector representatives. Workshops and analysis were guided by Normalisation Process Theory, the Implementation STakeholder Engagement Model (I‐STEM) and Meleis' Transitions Theory. Data were analysed using reflexive thematic analysis and collaborative synthesis.

**Findings:**

Stakeholders identified a shared need for a simple, conversation‐based tool that would help caregivers articulate their needs, receive timely information and build confidence in their role. The resulting toolkit comprises five co‐produced tools underpinned by a set of key principles, ranging from early admission resources to post‐discharge supports, all designed to support personalised, caregiver‐inclusive discharge planning. Participants emphasised the importance of respectful dialogue, a named point of contact, and clarity about post‐discharge support. Yet, variation in local infrastructure and workforce capacity remain barriers to implementation.

**Conclusion:**

A personalised discharge toolkit co‐designed with caregivers and professionals can strengthen transitional care and promote caregiver preparedness. Embedding this prototype into routine hospital practice will require organisational support, digital readiness and sustained attention to equity and inclusion.

**Patient or Public Contribution:**

This study was co‐produced through a participatory action research (PAR) approach involving individuals with lived experience of caregiving, alongside health and social care professionals and voluntary sector representatives. Informal caregivers actively shaped the design, delivery and iterative development of the personalised discharge planning toolkit across four co‐design workshops. Their insights informed key themes including recognition of caregiver identity, communication challenges and post‐discharge support needs. Caregivers contributed to analysis and interpretation of findings through discussion, feedback and validation during and after workshops, ensuring the toolkit was grounded in real‐world experiences and addressed the complexities of caregiving transitions.

## 1 Background

Hospital discharge is a pivotal moment in a patient's care journey, particularly for those with complex needs and their informal caregivers. In this paper, the term informal caregiver refers to family members, friends or others who provide unpaid support to someone with health or social care needs. We use the term inclusively, recognising that not all individuals self‐identify as ‘carers’ and that caregiving roles are often fluid and contested. For clarity and consistency across an international readership, we apply ‘informal caregiver’ throughout to encompass all those providing unpaid care, regardless of whether they adopt this label themselves.

Effective planning at discharge supports continuity of care, reduces readmissions and alleviates stress for both patients and caregivers [1]. The present study focuses on transitional care, a broader concept that includes both discharge planning and the post‐discharge period, emphasising safety, coordination and ongoing support [2, 3, 4]. Despite increased policy focus, informal caregivers are still frequently excluded from discharge planning, even though transitional care interventions have been shown to reduce healthcare use and improve outcomes [5, 6]. As a result, caregivers often feel unprepared for responsibilities such as medication management, mobility support, wound care and emotional reassurance [7], leading to fragmented care, increased burden and poorer outcomes [8]. This burden is particularly acute during transitions, especially when families receive little support in navigating long‐term care arrangements [9].

US models such as IDEAL promote early caregiver engagement and clearer communication, but their uptake has been inconsistent even in their original context [10]. In the United Kingdom, discharge practices continue to be driven more by organisational efficiency than by personalisation or collaboration [11], with communication breakdowns and local variation compounding these challenges [12].

Person‐centred and personalised care emphasise trust, shared decision‐making, and attention to goals, values and social context [13, 14], with cultural humility, contrasting with earlier notions of cultural competence, now recognised as a lifelong process of reflection, power‐sharing and responsiveness to diversity [15, 16, 17, 18]. This shift moves away from static notions of competence towards a relational and equity‐focused approach, which aligns closely with the principles of personalised care and the relational model underpinning our prototype toolkit. While these values are embedded in policy, they are inconsistently applied in practice. Barriers include time constraints, a lack of standardised tools, and persistent inequities, particularly for marginalised groups [8, 19].

Meleis' Transitions Theory explains why this matters: changes in roles, identities and expectations can leave caregivers distressed and unsupported and at greater risk of harm [2, 3, 4]. Evidence shows such stress effects caregivers' mental health, making support at discharge vital [20]. Discharge planning must therefore provide emotional and structural support, not just logistical coordination. Transitions Theory provides a useful interpretive lens for understanding our findings; by highlighting the relational and systematic aspects of transition we can identify discharge as a process that requires preparation, communication and collaboration.

Structured interventions such as CSNAT‐I show benefits but remain confined to palliative care and assume a fixed caregiver role [21, 22]. Our PROSPERO‐registered rapid review [23] found no co‐produced, scalable models for wider settings. This study addresses that gap through a participatory action research (PAR) approach to co‐design a personalised discharge toolkit. Working with caregivers, professionals and voluntary sector partners, we aimed to create a practical framework to support continuity of care and better prepare caregivers for their role at home.

## 2 Methods

### 2.1 Study Design

We used a PAR design to co‐produce a personalised hospital discharge toolkit, focusing on informal caregiver involvement in transitional care. Our goal was to develop a practical tool to support personalised, caregiver‐inclusive discharge.

This study built on three prior phases (2021–2023): a PROSPERO‐registered rapid review (CRD42022337444), stakeholder consultations and qualitative research exploring barriers to caregiver involvement. These activities identified key principles and promising features for workshop exploration. Earlier phases also included interviews and survey responses from a wider range of caregivers, which informed workshop design and analysis.

The study drew on Normalisation Process Theory (NPT) [24, 25], which explains how practices embed through sense‐making, relational work and integration. We also applied Meleis' Transitions Theory [2, 3, 4] through the analysis, which identifies discharge as a psychosocial shift in roles and identities, often leaving caregivers unprepared. Co‐production centres caregiver experience in designing practical and emotional support for this transition.

To guide inclusive stakeholder engagement, we used the Implementation STakeholder Engagement Model (I‐STEM) [26], which outlines five domains: objectives, stakeholder mapping, engagement approaches, qualities and outcomes (Appendix 1). The project was led by a multidisciplinary team with expertise in implementation science, transitions theory and participatory methods. Caregivers, professionals and voluntary sector partners contributed throughout, shaping the focus, designing workshops and co‐developing toolkit content. Our approach reflected calls to embed qualitative evidence into policy and service design [27] and the participatory ethos shaping commissioning and improvement practices [28]. At the same time, we remained alert to critiques of power and voice in co‐production [29, 30].

### 2.2 Participant Recruitment, Characteristics and Facilitators

We used purposive sampling to recruit individuals with personal or professional experience of hospital discharge. Participants were identified through caregiver organisations and professional networks. Recruitment was supported by collaborators across the NHS, voluntary sector and academic team.

All four workshops were conducted online using the software platform Blackboard Collaborate. Digital access support was offered where needed to support inclusive participation.

In total, 14 participants took part in the co‐design process, representing:

- **Caregiver reference group (*n*** = **4):** Individuals with lived experience of caring for a family member or friend.
- **Healthcare professionals (*n*** = **6):** Primarily working in hospital care, including physiotherapists, discharge coordinators and nurses, with links into community care through discharge liaison roles. No medical staff (e.g., residents, registrars and consultants) were recruited, reflecting both the focus of discharge coordination within nursing and allied health teams and practical recruitment constraints.
- **Voluntary sector representatives (*n*** = **4):** Involved in post‐discharge community care and caregiver support.

Eight members of the research team facilitated the workshops, contributing methodological and clinical expertise. Their role was to design and guide the activities and to support inclusive discussion. They did not contribute data as participants.

Each workshop involved 10–12 stakeholders in total, allowing for diverse perspectives across caregiver, clinical and community domains. This sample size is consistent with established norms for co‐design research [31].

### 2.3 Data Collection

Data were collected through four online co‐design workshops held in 2023. Workshops were facilitated by the research team using structured templates and visual tools.

Materials included field notes, transcripts, Jamboard outputs, annotated templates and post‐it content. Summary documents were shared after each session to support iterative learning and refine ideas.

Member checking was embedded throughout. Preliminary findings were presented to participants, Voluntary Organisations Network North East (VONNE) project team, and academic leads for feedback, strengthening trustworthiness and grounding findings in stakeholder experience.

### 2.4 Workshop Structure and Toolkit Development

Four workshops were designed with distinct goals, building iteratively on each other. Outputs from one session informed the next, enabling real‐time refinement of the toolkit.

- **Workshop 1:** Explored lived/professional experiences of discharge and defined ‘good practice’.
- **Workshop 2:** Generated toolkit ideas using visual prompts and themes.
- **Workshop 3:** Reviewed a draft toolkit, with detailed feedback on tone, content and accessibility.
- **Workshop 4:** Refined the revised draft, focusing on structure, language and feasibility.

Between sessions, the research team synthesised outputs and incorporated participant feedback into ongoing design. Member checking occurred continuously.

**Figure 1:**
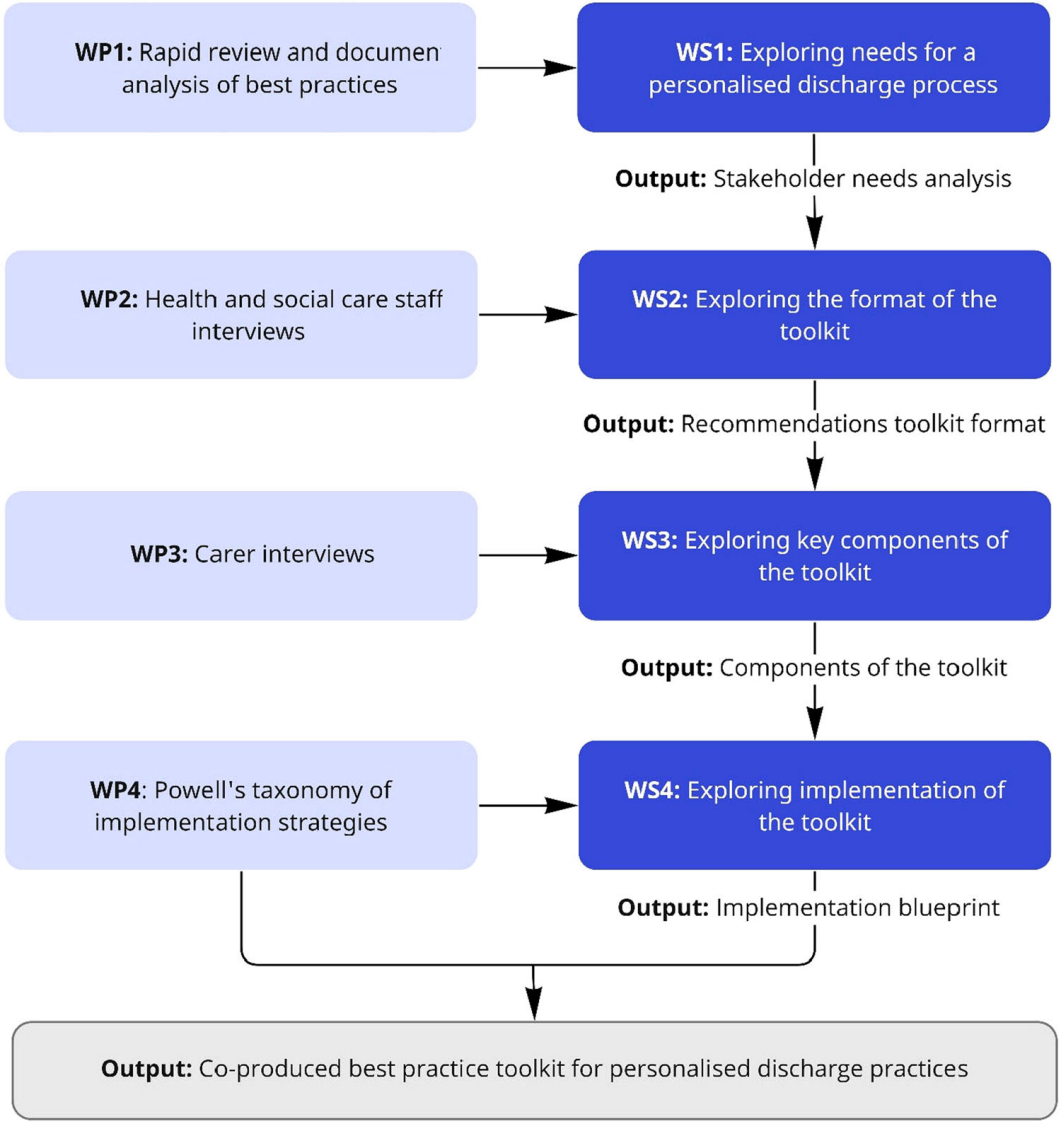
Workshop design and toolkit production process.

The workshops generated draft materials, which were iteratively refined into a prototype toolkit comprising five tools and a set of underpinning principles. Figure 1 summarises the design process and progression across the four workshops.

### 2.5 Ethics and Consent

Ethical approval for the study was granted by University of Northumbria at Newcastle Ethics Committee (Ref: Ref: 42061). All participants received detailed information about the study and gave written informed consent before taking part.

### 2.6 Data Analysis

We used reflexive thematic analysis [32, 33, 34], chosen for its focus on subjectivity, reflexivity and meaning co‐construction, aligning with the participatory ethos. Our goal was to identify stakeholder‐grounded themes to support toolkit development.

Data (transcripts, field notes, Jamboards and summaries) were imported into NVivo. Researchers met to discuss coding and interpretations, refining the framework and resolving discrepancies. The coding framework was then applied and iteratively refined.

NPT underpinned the wider programme of research, providing a framework for considering the implementation and normalisation of new practices [24, 25], using concepts like coherence and collective action to assess feasibility and relevance. In revising and interpreting the findings, we also drew on Meleis' Transitions Theory [2, 3, 4], which illuminated the multiple and overlapping role shifts experienced by caregivers during discharge.

Toolkit constructs were derived from this analysis and are presented in Table 1. Interpretation was recursive, supported by research team discussions and participant feedback.

**Table 1:** NPT constructs, themes and key insights.

NPT construct	Co‐produced theme	Key insights from workshops
Coherence	Recognition of caregiver status	Lack of shared understanding about who is the ‘caregiver’; invisible roles; differing expectations
Cognitive participation	Discharge planning as a co‐produced practice	Caregivers not routinely involved or ‘invited in’; professionals unaware of carer perspectives
Collective action	Infrastructures, systems and ways of working	Fragmented IT systems; lack of dedicated discharge roles; implementation pressures
Reflexive monitoring	Supporting caregivers' needs post‐discharge	Feedback loops missing; lack of follow‐up contact; need for post‐discharge points of contact

## 3 Findings

Here we present the findings from the co‐design process. Constructs were developed through collaborative analysis of workshop materials and aligned to draft components of the toolkit (see Table 1). These themes reflect what stakeholders saw as necessary to support meaningful, personalised discharge planning conversations. Figure 1 provides a visual overview of how participant input was captured and clustered.

In the following, quotes from the workshops themselves are noted as such. Quotes used in the co‐production workshops were originally drawn from earlier interviews and focus groups conducted during Work Package 3. These quotes were presented during workshops to stimulate discussion and co‐design activity and informed subsequent theme development.

Throughout this paper we use the term ‘caregiver’ for consistency and international clarity. Yet, we recognise that in the UK context the term ‘carer’ is more common, and we retain it in direct participant quotes where it reflects individuals' own language.

**Figure 2:**
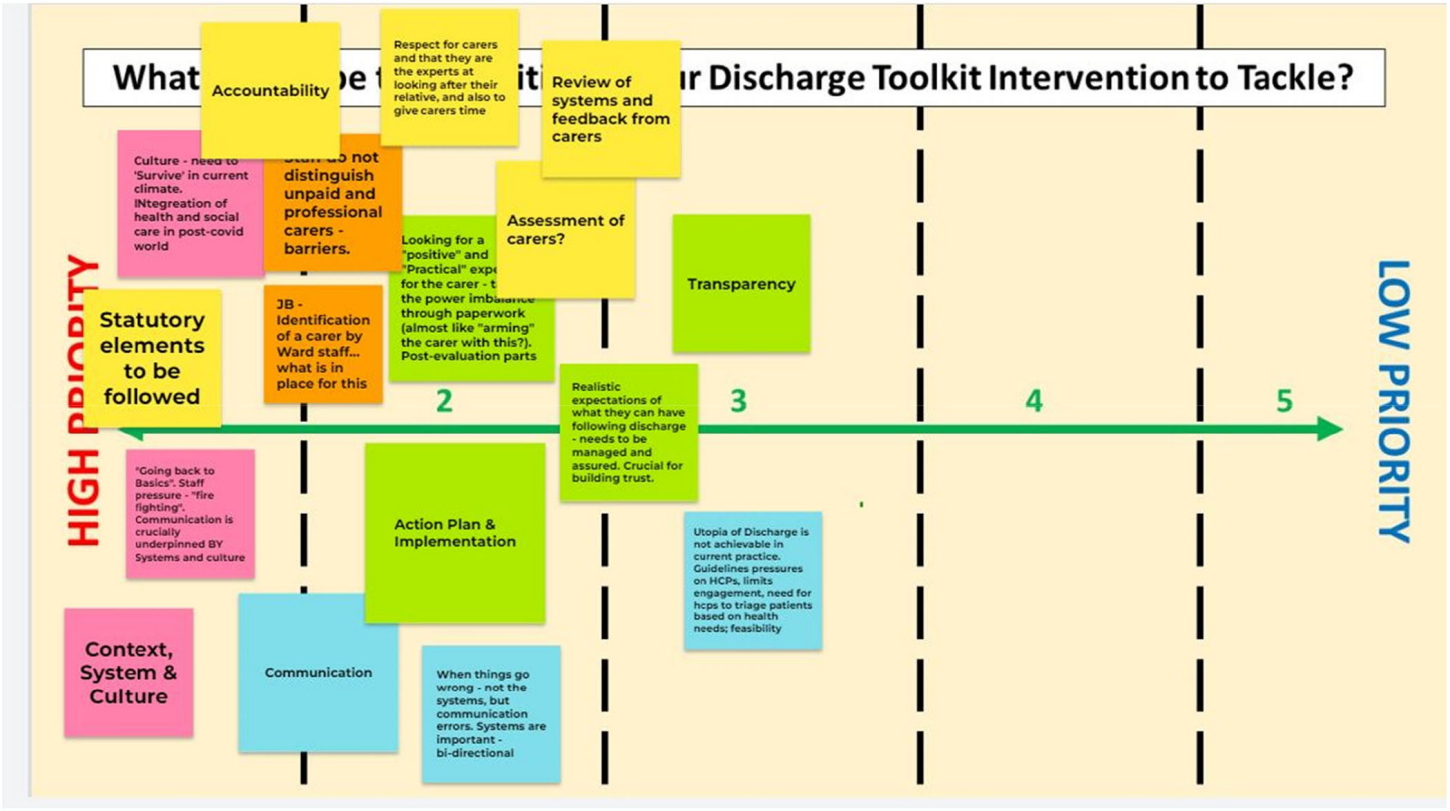
Excerpt from digital whiteboard (Jamboard).

An excerpt from the digital whiteboard (Jamboard) used in the workshops is available in Figure 2. These virtual whiteboards were used during co‐design workshops to capture real‐time input from stakeholders. This visual illustrates the collaborative approach and iterative nature of toolkit development. Identifying information has been removed.

For context, the co‐design process ultimately produced a prototype toolkit of five tools underpinned by shared principles, which we reference where relevant to illustrate how themes translate into practice.

### 3.1 Managing Stakeholder Expectations

The workshops repeatedly highlighted mismatches in expectations between caregivers and professionals as significant barriers to discharge planning. This mismatch in expectations represents a breakdown in shared sense‐making, an issue of ‘coherence’ in NPT terms.

Professionals should specifically ask carers what they need to feel confident to care for the patient at home.

Workshop 4, HCP

Caregivers expected partnership and shared responsibility, but these expectations were often unmet, leading to frustration and exclusion.

Carers don't need a process that is done to them. They should be equal partners. They need to know why the questions are being asked. And what realistically to expect will happen next.

Workshop 2, Jamboard

Conversely, professionals often presumed caregivers were fully capable of managing post‐discharge care without explicit verification or inquiry, leading to misjudgements about caregivers' readiness and support needs:

Start discharge planning on admission.

Workshop 1 13:52 chat log

Participants proposed solutions centred on early and clear communication. It was suggested that professionals initiate conversations at the earliest point of hospital admission to manage caregivers' expectations realistically. Such early dialogues were seen to mitigate misunderstandings and align expectations on both sides, facilitating smoother transitions from care.

### 3.2 Recognition of Caregiver Status

All stakeholder groups agreed a pervasive difficulty to initiating early effective discharge planning was accurately identifying the primary caregiver, defined here as the informal caregiver who assumed the lead role in coordinating or providing day‐to‐day care. This identification issue arose in part as discharge staff would mistakenly direct critical information towards family members who were not the primary caregiver, or who were not actively involved in daily care routines.

A persistent tension was the need to respect patient autonomy, even where patients would require substantial support post‐discharge. In some cases, this led to a default assumption that patients would manage independently, and a missed opportunity to assess and plan for informal caregiver involvement.

As one caregiver explained:

on admission, the ward asked my father lots of questions about himself, and he couldn't answer because he had Alzheimer's. If I'd been there, I could have started the ball rolling in answering all those question[s].

Workshop 2, chatlog

Participants suggested ‘Carer Passports’ to formalise caregiver identity, while acknowledging many of those providing care might not self‐identify as ‘(unpaid) carers’ and might use phrases such as ‘look after’ or ‘support’.

The lack of processes to formally identify or engage informal caregivers undermines their enrolment in the discharge process, limiting ‘cognitive participation’ in NPT terms. To address this, attendees recommended the appointment of dedicated staff to identify and support informal caregivers within hospital wards, recognising diverse caregiving identities without imposing a uniform ‘carer’ label.

### 3.3 Discharge Planning as a Partnership Practice

Participants unanimously stressed the importance of viewing discharge planning as a joint process, a partnership between caregivers and health professionals, which recognised the value of contributions to care.

One specific intervention proposed was a ‘welcome letter’ that could be delivered to caregivers on admission. This could facilitate early acknowledgement of discharge planning as a collaborative endeavour, highlighting caregivers' active role from the outset. The letter immediately positions caregivers as essential partners in discharge planning, explicitly recognising their skills, knowledge and lived experience, and affirming their personhood, ensuring their voices are genuinely heard and valued from the very start:

Carers are silent partners. Its [a] fight to be heard.

Workshop 2 Jamboard

Workshop discussions also surfaced power imbalances between caregivers and professionals, arising from unequal knowledge of healthcare systems. Professionals' greater familiarity with NHS systems sometimes unintentionally marginalised caregivers from important conversations:

Don't assume Carer understands hospital processes and language.

Workshop 2 Jamboard

Participants emphasised caregivers' empowerment as a necessary component of effective partnership in discharge processes. Caregivers' repeated calls to be treated as equal partners highlight the need for joint working and engagement; these are core elements of NPT ‘collective action’.

### 3.4 Infrastructures, Systems and Ways of Working

Systemic issues emerged prominently, notably how language barriers, particularly medical jargon and NHS terminology, posed real challenges for caregivers:

Avoiding medical jargon. Using clinical abbreviations can exclude patients/carers. Need explanation and clarity of what the different systems are called.

Workshop 1 Field notes

Systemic constraints such as workforce capacity and workload led to caregivers feeling neglected or undervalued. Transparency about these constraints was thought to manage caregiver expectations better and foster trust. Stakeholders recommended structured time points clearly dedicated to discharge planning conversations, explicitly communicated to caregivers.

Final toolkits developed needed to fit into this challenging system:

the toolkit needs to be user friendly [move beyond] academic tools which may prompt discussion, [so] ward staff having the time or the skills to do justice to these tools.

WK3 Jamboard 10.35

These systemic barriers limited sustainable ‘collective action’ in NPT terms.

### 3.5 Supporting Caregivers' Needs Post‐Discharge

A further finding across workshops was caregivers' uncertainty about where and how to access support for wider needs after discharge, including financial advice and caregiver assessments. Participants noted that caregivers often did not know what questions to ask professionals, limiting their ability to secure vital resources. One proposed solution was to provide caregivers with a structured list of core questions, such as ‘*What's preventing me from going home?*’, to empower them and prompt clearer communication. Participants also recommended establishing a single, clear point of contact post‐discharge to help caregivers navigate ongoing medical, social and financial queries. This single‐contact concept resonated strongly, as it was seen to reduce stress, confusion and the risk of preventable readmission.

Participants further reflected on how systemic and policy‐level changes, particularly during and after Covid‐19, had disrupted previously embedded practices that supported early engagement with families. One professional recalled:

‘I think what we lost [.] partly because of COVID and the new policy [.] is that each of our elderly wards had a dedicated social worker. And on admission to that ward the social worker would have contacted the family [.] the next of kin’.

WP2 slides—originally derived from professional interview, WP3

The absence of clearly assigned staff roles was frequently noted as a barrier to caregiver inclusion, particularly in the early stages of admission. Participants emphasised that responsibility and follow‐up mechanisms were critical for improving continuity of care. The proposal for follow‐up contacts and feedback mechanisms aligns closely with the NPT construct of ‘reflexive monitoring’. To interpret these themes through a theoretical lens, we mapped them onto the four core constructs of NPT. Table 1 summarises these alignments, illustrating how caregiver and professional experiences reflect recognised enablers and barriers to embedding new practices. This mapping provided the foundation for developing the prototype toolkit, described in Section 3.6.

### 3.6 Toolkit and Implementation Blueprint

Building on this theoretical mapping, the prototype toolkit was structured to directly target the barriers identified in analysis. Each tool was designed to address a specific gap in discharge practice while collectively reinforcing principles of partnership, clarity, responsiveness and continuity. Table 2 summarises how the toolkit components map onto these barriers and illustrates their intended function in practice. The full toolkit has been published on University of Northumbria Figshare repository [35], ensuring open access for testing and adaptation.

**Table 2:** Toolkit components, the barriers they address, and how they work in practice.

Toolkit tool	Barrier/theoretical issue addressed	How it addresses in practice
Welcome letter	Lack of orientation and role clarity; caregivers often excluded from early discharge discussions (NPT: coherence, legitimation).	Provides plain‐language orientation from admission; clarifies caregiver role and expectations; reduces uncertainty.
Admission conversation guide	Caregivers' knowledge not consistently recognised; professionals uncertain how to involve caregivers (NPT: cognitive participation).	Prompts staff to invite caregiver knowledge of routines, risks and home context from day one; validates caregiver identity.
'What matters to you’ prompt card	One‐way information flow; caregivers' priorities sidelined in clinical processes (Transitions theory: unacknowledged role shift).	Simple, repeatable cue for caregivers to articulate priorities/questions; normalises two‐way communication.
Discharge planning checklist	Fragmented planning; important domains omitted; multidisciplinary discussions inconsistent (NPT: collective action).	Structures discharge discussions; ensures coverage of medications, equipment, supports and transport; increases transparency.
Post‐discharge contact card	Lack of continuity after discharge; caregivers unsure where to seek help; safety‐netting gaps (NPT: reflexive monitoring).	Provides named contact and ‘what to do if…’ guidance; supports continuity and reduces avoidable readmissions.
Underlying principles	Systemic issues of exclusion, inconsistency and opacity across discharge practices.	Partnership, clarity, responsiveness and continuity frame the intended use of all tools in practice.

*Note:* Together, these tools represent a prototype resource that translates co‐design insights into practical components, providing the basis for the discussion of feasibility, equity and future implementation that follows.

## 4 Discussion

### 4.1 Overview of Key Findings

This study identified five interlinked barriers to personalised and caregiver‐inclusive discharge planning: mismatched expectations between professionals and caregivers; inconsistent recognition of caregiver status; limited use of co‐produced approaches; systemic and infrastructural constraints; and insufficient post‐discharge support. Current processes, while aligned with policy, too often fail to position caregivers as active participants.

The co‐produced toolkit presented here responds to these challenges by offering a practical, relationally grounded model that centres caregivers as equal partners in discharge planning. Its feasibility, however, is shaped by wider organisational and cultural conditions, including time pressures, fragmented digital systems and entrenched power asymmetries.

Participants also reflected on the disruptive impact of Covid‐19. Pandemic restrictions removed previously embedded practices that supported early family engagement, such as ward‐based social worker contact, and constrained caregiver visibility. These observations echo emerging evidence that Covid‐19 disrupted continuity of care for older patients and limited family involvement through visiting restrictions [36, 37, 38]. Together, these findings emphasise the timeliness of interventions that re‐establish caregiver engagement at crucial transition points.

The following sections situate our findings in relation to existing interventions (e.g., CSNAT), theoretical frameworks (NPT and Transitions Theory), and wider concerns around equity, caregiver identity and systemic inertia, before considering implications for implementation and measurable outcomes.

### 4.2 Interpreting Findings Through NPT

These themes also map meaningfully onto the four core constructs of NPT, providing a conceptual lens for understanding both the barriers to and opportunities for embedding personalised discharge practices. For caregivers in particular, ‘coherence’, a shared understanding of roles, expectations and discharge processes, was the most consistently raised concern, underpinning their ability to engage meaningfully in planning and post‐discharge care. The mismatch in expectations reflects a lack of ‘coherence’, a shared understanding of roles and processes, while difficulties in identifying and engaging caregivers point to a breakdown in ‘cognitive participation’. Systemic constraints and variable infrastructure limit ‘collective action’, and the absence of structured follow‐up impedes ‘reflexive monitoring’. This framing clarifies why personalised discharge is difficult to implement and what might support its sustainability.

While all stakeholder groups recognised barriers across the four NPT constructs, their emphasis differed. Caregivers predominantly raised concerns related to ‘coherence’ and ‘reflexive monitoring’, highlighting the importance of shared understanding and structured follow‐up. In contrast, health and social care professionals focused more on ‘cognitive participation’ and ‘collective action’, reflecting challenges in integrating new approaches within established workflows and time constraints. For professionals, ‘cognitive participation’ was shaped by whether the intervention could be legitimised within routine practice, especially amid high workloads and system pressures. These differing emphases underscore the relational complexity of embedding personalised discharge planning across fragmented systems.

This alignment strengthened the analytical link between workshop themes and NPT constructs.

### 4.3 Operationalising Person‐Centred and Personalised Care

To demonstrate how the co‐produced toolkit responds directly to priorities surfaced through the workshops, Table 3 maps each thematic domain (expectation management, caregiver identity, co‐production, systemic context and post‐discharge support), onto relevant toolkit components. These components were designed to be practical, user‐friendly and adaptable, supporting overstretched staff while empowering caregivers.

**Table 3:** Mapping toolkit components to thematic priorities and underlying principles.

Theme	Toolkit component(s)	Intended function(s)	Underlying principle(s)
1. Managing stakeholder expectations	Welcome letter on admission	To clarify roles and support needs early and prevent mismatched assumptions between caregivers and professionals.	Early engagement (PCC); clear communication (PCC); role clarity and anticipatory planning (Personalised care)
	Early structured conversations using the toolkit		
2. Recognition of caregiver status	Caregiver Passport (supplementary mechanism)	To ensure the correct person is identified and included in planning, particularly where carer identity is often invisible or unacknowledged.	Recognition of relational identity (PCC); inclusive, tailored engagement strategies (Personalised care)
	Named point of contact during and after discharge		
3. Discharge planning as co‐produced practice	Jointly completed toolkit sections	To support partnership‐based planning and elevate caregiver contributions in discharge decision‐making.	Shared decision‐making and empowerment (PCC); personalised support planning (Personalised care)
	Toolkit used as a conversation tool during stay		
4. Infrastructures, systems and ways of working	Toolkit designed to be jargon‐free and concise	To ensure toolkit usability in time‐pressured settings and improve communication through clarity and integration.	Accessibility and clarity of communication (PCC); integration into workflows for feasibility (Personalised care)
	Embedded into existing documentation or electronic systems		
	Includes prompt questions for professionals		
5. Supporting caregivers post‐discharge	Simple structured question list	To offer continuity of support, reduce post‐discharge uncertainty and mitigate readmission risk.	Continuity of relationships and communication (PCC); structured, tailored follow‐up (Personalised care)
	Signposting section		
	Follow‐up mechanism		

The design was explicitly informed by principles of person‐centred care (PCC) and personalised care. While overlapping in intention, PCC emphasises relationships, dignity and respect, whereas personalised care focuses on tailoring support, informed choice and shared planning [13, 14]. Embedding both approaches was seen as necessary to bridge the persistent gap between policy and the realities of discharge practice, supporting continuity and inclusion of caregivers.

Conceptualisations of PCC within healthcare organisations, however, vary considerably. Some staff adopt well‐aligned relational definitions, while others interpret PCC so broadly that it risks becoming a vague or symbolic gesture, undermining meaningful implementation [39, 40]. Evidence suggests that person‐centred approaches are associated with improved patient outcomes, caregiver experiences and service efficiency, but their success depends on consistent, clear application in practice [41].

At the same time, it is important to acknowledge that caregiver and patient perspectives are not always aligned. In some circumstances, particularly where patients experience cognitive decline or communication difficulties, strengthening caregiver involvement can risk overshadowing the patient's own preferences. This tension has been well documented in dementia research, where caregivers' advocacy may unintentionally minimise patient autonomy [42, 43]. For this reason, the toolkit should be understood as a prototype resource that aims to support collaborative conversations, rather than a guaranteed route to genuine person‐centredness. Future iterations will need to pay close attention to how caregiver involvement can complement, rather than displace, patient voices in discharge planning.

By embedding both person‐centred and personalised care principles into the toolkit, and by foregrounding inclusive, co‐produced design, this study ensures that values of dignity, flexibility and authentic collaboration are embedded in practical, scalable ways into discharge planning practices.

### 4.4 CSNAT/Toolkit

The co‐designed discharge toolkit developed in this study offers a flexible, inclusive approach to caregiver involvement, but faces real‐world implementation challenges. Comparisons with the Carer Support Needs Assessment Tool (CSNAT) are instructive. Originally developed in palliative care, CSNAT demonstrated strong potential for identifying and addressing caregiver needs [44] but has had limited uptake beyond specialist contexts due to complexity and resourcing challenges [22]. Workshop participants raised similar concerns about our toolkit, noting systemic pressures, high workloads and limited staff time, that risk undermining any structured intervention. To address this, they recommended embedding the toolkit into existing workflows and IT systems, introducing it at admission alongside welcome materials, and assigning responsibility to a clear role such as discharge coordinator or caregiver liaison.

Unlike CSNAT, which assumes a clearly defined caregiver role, our toolkit accommodates the diversity and fluidity of caregiving. It recognises that many individuals do not self‐identify as carers (see Section 3.2) and allows flexible engagement that respects self‐identification and preferences. Mechanisms such as a Carer Passport could further strengthen visibility and recognition of caregiving roles within discharge planning. Another key distinction is emphasis on early partnership: tools like the Welcome Letter position caregivers as contributors from the outset rather than recipients of support at crisis points, supporting relational rather than transactional care [13].

Uptake will require cultural and structural change, particularly recognising caregivers as equal partners from admission, not as passive recipients of discharge plans (see Section 3.3). Future work should examine which components are most pertinent and how best to embed them without increasing staff burden.

In brief, while CSNAT remains a valuable comparator, our toolkit offers a more flexible, identity‐sensitive and relationally grounded approach to caregiver support in hospital discharge.

### 4.5 Recognising Caregiver Identity Complexity Through Meleis' Transitions Theory

The explicit recognition of caregiver identity complexity, as highlighted by stakeholders in our co‐produced discharge toolkit, resonates strongly with Meleis' Transitions Theory [2, 3, 4]. According to Meleis, transitions are periods marked by shifts in roles, identities, expectations and responsibilities. These transitions, such as hospital discharge, are experienced uniquely by individuals depending on their perception of their evolving roles and identities. Effective support must therefore be personalised and attuned to these experiences of transition.

Importantly, Meleis [3, 4] demonstrates that successful role transitions depend upon clear, supportive communication that resonates authentically with the transitioning individuals' self‐understanding and identities. The finding from our workshops, that many individuals fulfilling caregiving responsibilities do not identify with the term ‘carer’, has clear implications here. If discharge planning interventions rigidly assume a caregiver identity, they risk misalignment with caregivers' self‐perception, potentially alienating or marginalising them, thereby undermining the quality of transitional support.

In contrast, our co‐produced toolkit explicitly foregrounds the need for adaptable language and flexible engagement approaches. Participants strongly advocated that professionals should recognise and validate the diverse identities caregivers bring to the care situation, such as being spouses, parents, children or friends. This aligns with Meleis' emphasis on ‘meaningfulness’ as a critical attribute of successful transitions [45]. By explicitly acknowledging and affirming caregivers' personal identities, rather than imposing an external ‘carer’ identity, transitional interventions become more meaningful, accessible and likely effective.

Further, our approach directly contrasts with assessment tools like CSNAT, which, despite their structured practicality, implicitly assume participants' willingness and ability to identify as caregivers. This assumption, although subtle, risks creating barriers to engagement precisely at a moment when individuals face profound role and identity challenges. Meleis' theory emphasises that if identity incongruence persists, transitions can become stressful, maladaptive and even detrimental to health and well‐being [4, 45].

Given this, the integration of caregiver identity complexity within discharge planning interventions is imperative. By explicitly recognising and accommodating diverse caregiver identities, our co‐produced toolkit operationalises core theoretical insights from Meleis' work, providing more authentic, inclusive support during the critical transition from hospital to home.

### 4.6 Implementation, NPT and Professional Perspectives

NPT [34] provides a useful framework for assessing the implementation potential of the co‐produced toolkit. It highlights four domains that shape adoption: coherence (clarity of purpose), cognitive participation (stakeholder buy‐in), collective action (fit within everyday routines), and reflexive monitoring (capacity for evaluation and adaptation).

The toolkit engages with each of these domains by clarifying discharge expectations early in the hospital stay, fostering stakeholder ownership through co‐production, emphasising usability in time‐pressured environments, and supporting local adaptation. Embedding depends on alignment with workflows, training and sustained organisational support.

Professionals stressed that the toolkit must be practical, scalable and seamlessly integrated. They recommended introducing it at admission, embedding it in electronic records and assigning responsibility to designated roles (e.g., caregiver liaison) to ensure consistent use and to identify caregivers who might otherwise be overlooked. Integration and ownership were seen as critical to avoiding additional administrative burden.

Yet feasibility remains contingent on wider system readiness. Digital infrastructure and workforce capacity vary across NHS Trusts and community providers, with persistent fragmentation in data sharing and continuity of care [46, 47]. Media reports have similarly highlighted delays in digitisation, ongoing reliance on paper records, and bottlenecks in coordination [48]. National reviews continue to warn of unsafe discharge practices and unclear accountability [49]. These structural deficits constrain the normalisation of caregiver‐centred tools.

Stakeholders also highlighted the need for training in empathetic communication and personalised conversations. Current discharge practice often focuses narrowly on clinical instructions rather than enabling caregivers to articulate their own questions or needs. Reflective training and structured tools were seen as essential to support more relational discharge planning. Aligning implementation strategies with NPT therefore requires attention not only to organisational systems but also to the lived realities of frontline practice.

### 4.7 Caregiver Inclusion, Equity and Future Evaluation

Beyond structural readiness, inclusion also depends on recognising diverse caregiver identities and ensuring equity in toolkit rollout. Recognising diverse caregiver identities is central to inclusive discharge planning. Not all individuals providing care identify with the term ‘carer’; some prefer ‘care partner’, while others see their role as part of an existing relationship (e.g., spouse, sibling and friend) rather than a distinct identity [50]. Scholars have argued that describing this labour as ‘informal’ misrepresents its complexity and responsibility, with some contending there is ‘nothing informal about caregiving’ [51]. Caregiving, even when unpaid, is formal in its intensity and impact. A recent scoping review highlights the existential toll of caregiving and the importance of meaning‐making interventions to support identity shifts and role adaptation [52]. We therefore use ‘caregiver’ across this paper as the most inclusive term available, while recognising the limitations and exclusions inherent in any single label. This sensitivity to language informed both the co‐production of the toolkit and its emphasis on validating diverse caregiver roles.

Equity is also essential to the toolkit's future adoption. Our co‐design process engaged a range of caregivers and professionals, but under‐represented ethnically diverse caregivers, younger caregivers, and those facing language or cultural barriers. Future rollout should incorporate explicit equity strategies, including targeted outreach to seldom‐heard groups, translated and accessible toolkit materials, and co‐production with voluntary and community organisations that hold trusted relationships with marginalised populations. Embedding culturally tailored communication and interpreter support will be critical to ensuring accessibility and fairness across health and social care contexts.

These considerations cannot be separated from wider systemic challenges. Caregiving involves relational labour, emotional, organisational and embodied work that is often undervalued or rendered invisible in institutional contexts [53]. This invisibility sustains entrenched power asymmetries between caregivers and professionals, repeatedly identified across our workshops. Toolkit components such as simplified structured questions were designed to redress these disparities by supporting caregivers to articulate needs and participate as equal partners. Yet systemic inertia within healthcare [54] constrains the normalisation of caregiver‐centred tools. Without addressing these cultural and institutional barriers, co‐produced interventions risk being undermined and their transformative potential limited.

### 4.8 Limitations

This study has several limitations. First, the co‐design workshops involved a relatively small number of participants and may not capture the full diversity of caregiver perspectives, particularly those from under‐represented communities. A further limitation is that only four caregivers participated in the co‐design workshops. Their input was highly valuable but inevitably limited in scope. However, the co‐design process was situated within a broader programme of work: earlier work packages included interviews and survey responses from a wider range of caregivers, whose insights informed the design of the workshops and provided a foundation for analysis. Themes raised by the four caregiver participants were consistent with these earlier findings, suggesting wider resonance. Even so, the toolkit should be understood as a prototype resource that now requires further refinement and testing in practice, with larger and more diverse caregiver groups.

Second, and most notably, we were unable to include young caregivers in the co‐design process. This is a significant limitation, as young caregivers often face high levels of unmet need, are less likely to be recognised by professionals, and may have limited capacity to self‐advocate during discharge planning. Future iterations of the toolkit should prioritise the inclusion of this group to ensure its relevance and accessibility across the full spectrum of caregiving roles.

### 4.9 Future Research and Measurable Outcomes

Future evaluation of the toolkit should assess not only its feasibility in routine use but also its impact on measurable outcomes for both caregivers and patients. Relevant indicators may include hospital readmission rates, length of stay and delayed discharge episodes, alongside caregiver‐focused measures such as preparedness for caregiving, confidence in navigating services and satisfaction with discharge planning. Standardised tools, including validated caregiver preparedness scales, can provide comparable data across settings. Embedding these measures into evaluation will help determine whether the toolkit translates into improved continuity of care, reduced system strain and enhanced caregiver well‐being.

## 5 Conclusions

We have presented a co‐produced caregiver toolkit, developed as a prototype resource to support person‐centred and personalised approaches to discharge planning through collaborative conversations. It emphasises flexible caregiver identities, early partnership engagement and realistic implementation strategies; the toolkit offers a practical and inclusive way to improve transitions from hospital to home. Developed through iterative co‐production workshops, it is grounded in stakeholder insights and tailored for use in real‐world practice. Future research and implementation efforts should now build on this foundation, ensuring its principles are embedded sustainably in healthcare settings and that the rhetoric of partnership is matched by the lived reality of caregiving.

## Author Contributions

**Kathryn McEwan:** data curation, writing – original draft, writing – review and editing. **Tom Sanders:** conceptualisation, methodology, formal analysis, data curation, writing – original draft, writing – review and editing, project administration, funding acquisition. **Sue Carr:** conceptualisation, methodology, investigation, writing – review and editing, project administration, funding acquisition. **Peter Van Der Graaf:** investigation, writing – review and editing. **Susan Jones:** investigation, writing – review and editing. **Maria Raisa Jessica Aquino:** investigation, writing – review and editing. **Mitchell James Hogg:** formal analysis, investigation, writing – original draft, writing – review and editing. **Rakhshanda Hameed:** investigation, writing – review and editing. **Frank Lai:** writing – review and editing. **Christina Cooper:** writing – review and editing. **Sebastian Potthoff:** conceptualisation, methodology, formal analysis, data curation, writing – original draft, writing – review and editing, project administration, funding acquisition.

## Conflicts of Interest

The authors declare no conflicts of interest.

## Supporting information

Discharge_Caregivers_HEX_Appendix One_I‐STEM_11.06.25.

## Data Availability

The datasets generated and/or analysed during the current study are not publicly available due to confidentiality agreements with participants, but anonymised excerpts are available from the corresponding author upon reasonable request.
