# Supplementary material for: Co‐Producing Personalised Discharge Planning: Developing a Toolkit to Improve Caregiver Involvement in Hospital Transitions
Source: Health Expect. 2025 Nov 5;28(6):e70483. doi: 10.1111/hex.70483 (PMC12589894; doi:10.1111/hex.70483)
Supplement: Supplementary file 1 — Discharge_Caregivers_HEX_Appendix One_I‐STEM_11.06.25. [file HEX-28-e70483-s001.docx]

Planning and evaluating stakeholder engagement in the co-production of a discharge from hospital toolkit: Application of the I-STEM model

Aims:

- To co-produce a discharge from hospital toolkit based on the benefits of a personalised care model
- To co-produced tailored strategies to promote the implementation of the toolkit

Methods:

- Participatory research approach
- Four online co-production workshops

Participants

- Carer representatives
- Trust staff
- Voluntary sector staff

Possible Objectives for Engaging Stakeholders in Implementation Work

**Summary**: Co-production workshops will aim to ***understand*** participants’ needs with regards to delivering and receiving personalised discharge practices. This will involve using participatory research methods to ***access*** participants’ expertise and experiences. We also aim to get ***access*** to documents pertaining to discharge processes within the chosen Trusts. As part of this process, we will ***verify*** the emerging findings from the qualitative research carried out alongside the workshops. As a by-product of these activities we hope to ***inform*** participants about examples of best practice, as well as gaps in the quality of care provided within the Trust. We hope to ***enrol*** stakeholders and make them active participants in the co-production of the toolkit.

| **ENGAGEMENT OBJECTIVES** | |  |
| --- | --- | --- |
| Focus | Description | Application |
| Inform | Objective of engagement activities may focus on serving to inform and raise stakeholders’ awareness of the innovation. | Inform participants about best practice examples in the region (document analysis) and further afield (rapid review). |
| Understand | Objective of engagement activities may focus on impacting on implementers’ understanding of implementation related issues. | Understand stakeholders’ needs with regards to delivering and receiving personalised discharge practices.  Understand barriers and facilitators to the implementation of the toolkit |
| Verify | Objective of engagement activities may focus on verifying implementers’ initial ideas about the implementation activities. | Verify emerging findings from qualitative research i.e., interviews and focus groups with carers and professionals.  Verify initial ideas (prototype) of a best practice toolkit. |
| Enrol | Objective of engagement activities may focus on serving to enrol stakeholders in the implementation process. | Enrol participants in the production of a best practice toolkit to facilitate acceptability, adoption, and integration of the toolkit. |
| Access | Objective of engagement activities may focus on impacting on financial or material resources available for the implementation work. | Access participants expertise and experiences with regards to the hospital discharge process  Access relevant policy documents, practice guidance, quality improvement/service evaluations, re-admission documentation, repeat admission documentation. |
| Do | Objective of engagement activities may focus on practically doing elements of the implementation work. | Develop the purpose, format and content of the toolkit  Tailor strategies to address local implementation barriers  Disseminate the toolkit across all acute NHS FTs in the NE and Yorkshire region |

Possible Reasons for Choosing Stakeholders for Implementation Activities

**Summary**: It will be important to recruit participants with ***expertise*** in delivering and receiving discharge practices. By including both those who deliver and those who receive discharge practices we will achieve a productive exchange of ideas and expertise, which will increase the acceptability and usefulness of the developed toolkit. One concern is that there may be a lack of ***trust*** amongst participants from diverse groups. Carers may have negative and even traumatic experiences which they may not feel comfortable to share. Equally professionals may feel attacked if confronted with negative reports about discharge practices. Therefore, it will be important to address this issue heads on by setting clear ground rules of mutual respect where everyone feels safe to share their viewpoints.

| **STAKEHOLDER MAPPING** | |  |
| --- | --- | --- |
| Reason | Description | Application |
| Influence | The degree to which stakeholders have influence on the implementation of an innovation, either directly or via other stakeholders. | Discharge leads – will be pivotal in providing access to Trust staff. Will support implementation of the toolkit.  Voluntary sector organisations – support access to trusts. Support implementation. |
| Expertise | The degree to which stakeholders have information, counsel, or expertise relevant to the implementation of the innovation. | Carer representatives – lived experiences and real-life examples of good and poor discharge practices.  Voluntary sector organisations – representing counsel and expertise in supporting carers.  Trust staff – diverse experiences with regards to designing, delivering, and evaluating discharge processes |
| Orientation | The degree to which stakeholders’ views or attitudes towards the innovation are favourable or resistant. | Carer representatives – may have more negative attitudes due to previous experiences with discharge.  Voluntary sector – more neutral perspective but representing carers’ views.  Trust staff – probably mixed views and attitudes. |
| Impact | The degree in which the implementation project impacts on the stakeholder. | Carer representatives – impact on the discharge practices they will receive.  Voluntary sector – indirectly via their association with carers.  Trust staff – will be implementing and delivering the best practice guidance. |
| Capacity | The degree to which stakeholders have the capacity to take part in the engagement activity. | Trust staff – will they have the capacity to support the development / implementation of the toolkit? |
| Trust | The degree to which there is mutual familiarity and trust between the potential stakeholders. | A lack of trust between different stakeholder may be a potential barrier to this work. This is due to carers’ negative and sometimes traumatic experiences in hospital discharge processes. Participants may not feel save to share their experiences with the wider group due to such concerns around trust. Equally Trust staff may feel threatened or attacked if confronted with negative reports about discharge practices. Therefore, it will be important to address this issue heads on by setting clear ground rules of mutual respect where everyone feels safe to share their viewpoints. |

*Possible Ways of Working with Stakeholders when Implementing Change*

**Summary:** We chose ***collaborating*** as the most appropriate way of working with stakeholder to design and implement a discharge from hospital toolkit. By collaborating with relevant stakeholders, we will ensure that the developed toolkit will be tailored to both the stakeholders and contextual needs. This is turn will facilitate the update and integration of the developed toolkit. In this process we will ***assess*** the views and experiences of wider stakeholder groups and ***verify*** our preliminary findings with workshop participants. Once the toolkit has been produced with will be working with Discharge Leads and Voluntary Sector organisations to disseminate the toolkit across participating Trusts.

| **ENGAGEMENT APPROACHES** | | |
| --- | --- | --- |
| **Ways of working** | **Description** | **Application** |
| Disseminating | Disseminating involves giving out information about the innovation. | Once a toolkit has been produced, we need to disseminate it across all acute NHS FTs in the NE and Yorkshire region. Discharge Leads and Voluntary Sector Organisations will be important in this process. |
| Assessing | Assessing involves gathering information from stakeholders that is relevant to the implementation activity. | Assessing stakeholders’ views and experiences using qualitative interviews and co-production workshops. |
| Consulting | Consulting involves offering implementation related information to selected stakeholders to seek their feedback or advice. | Seek feedback and advice on emerging findings from qual work in the co-production workshops. |
| Collaborating | Collaborating involves working closely with stakeholders on a common objective relating to implementation. | Working closely and collaboratively with stakeholders to co-produce and implement the best practice toolkit. |

Possible Qualities of Stakeholder Engagement Work

**Summary**: A high level of ***preparedness*** will be key to the success of the co-production workshops. This will involve preparing and disseminating headline findings from the qualitative research in advance to the workshops to give participants time to reflect on the findings. Workshops will take place ***regularly*** (every fortnight) to ensure that the information stays fresh on participants’ minds. Given the short duration of each workshop (online 2 hours) there will be a high level of ***structure*** involving brief presentations, engagement activities and discussions. Stakeholder will be asked to be ***active*** participants in all the workshop activities. We will use a variety of engagement activities (e.g., whiteboards and sticky notes) to facilitate this. Throughout the planning and delivery of the workshops, ***accountability*** will be kept at a minimum meaning that participants are free to contribute to an extend that is acceptable to them.

| **ENGAGEMENT QUALITIES** | | |
| --- | --- | --- |
| **Qualities** | **Description** | **Application** |
| Preparedness | Engagement can vary in terms of the degree to which stakeholders are prepared for the activity. | High |
| Structure | Engagement can vary in the degree to which the activity is structured. | High |
| Activity | Engagement can vary in terms of how actively stakeholders are engaged in the implementation work. | High |
| Regularity | Some stakeholder engagement activities are carried out on a regular basis, whereas other activities are more sporadic. | High |
| Accountability | Engagement may vary in terms of the degree to which stakeholders are hold accountable for their contribution. | Low |

*Possible Outcomes of Stakeholder Engagement Work*

**Summary**: This stage of the model will help us evaluate whether our stakeholder engagement activities have helped achieve the objectives that we have set out at the start. For example, we could conduct pilot a prototype of the toolkit with stakeholders that were not part of the workshops to ***verify*** its workability. We could conduct a think aloud study to ***understand*** whether the toolkit sufficiently captures stakeholders’ needs.

| **ENGAGEMENT OUTCOMES** | | |
| --- | --- | --- |
| **Outcomes of engagement work** | **Description** | **Application** |
| Informing | Engagement activities may result in stakeholders being more informed about the innovation that is being implemented. | Did they become more aware of best practice examples / quality gaps in the region? |
| Understanding | Engagement activities may result in providing a better understanding of stakeholders’ needs, barriers and facilitators with regards to the innovation. | Did we identify and prioritise their needs with regards to delivering and receiving personalised discharge practices?  Do we understand barriers and facilitators to the implementation of the toolkit? |
| Verifying | Stakeholder engagement may serve to verify initial ideas relating to the implementation of an innovation. | Did we manage to verify emerging findings from qualitative research i.e., interviews and focus groups with carers and professionals?  Did we verify initial ideas (prototype) of a best practice toolkit? |
| Enrolling | Engagement activities may result in a desired outcome by enrolling relevant people in the process. | Did we enrol participants in the production of a best practice toolkit to facilitate acceptability, adoption, and integration of the toolkit? |
| Accessing | Stakeholder engagement may serve to access resources or expertise necessary for implementation. | Did we manage to access participants expertise and experiences with regards to the hospital discharge process?  Did we manage to access relevant policy documents, practice guidance, quality improvement/service evaluations, re-admission documentation, repeat admission documentation? |
| Doing | Stakeholder engagement may serve to practically undertake elements of the implementation work. | Did we manage to co-develop a best practice toolkit?  Did we manage to tailor strategies to address local implementation barriers?  Did we manage to disseminate the toolkit across all acute NHS FTs in the NE and Yorkshire region? |
